# Biosorption and Biomineralization of U(VI) by the Marine Bacterium *Idiomarina loihiensis* MAH1: Effect of Background Electrolyte and pH

**DOI:** 10.1371/journal.pone.0091305

**Published:** 2014-03-11

**Authors:** Fernando Morcillo, María T. González-Muñoz, Thomas Reitz, María E. Romero-González, José M. Arias, Mohamed L. Merroun

**Affiliations:** 1 Departamento de Microbiología, Universidad de Granada, Granada, Spain; 2 Institute of Resource Ecology, Helmholtz-Zentrum Dresden-Rossendorf, Dresden, Germany; 3 Structural and Civil Engineering Department, University of Sheffield, Sheffield, United Kingdom; Belgian Nuclear Research Centre SCK/CEN, Belgium

## Abstract

The main goal of this study is to compare the effects of pH, uranium concentration, and background electrolyte (seawater and NaClO_4_ solution) on the speciation of uranium(VI) associated with the marine bacterium *Idiomarina loihiensis* MAH1. This was done at the molecular level using a multidisciplinary approach combining X-ray Absorption Spectroscopy (XAS), Time-Resolved Laser-Induced Fluorescence Spectroscopy (TRLFS), and High Resolution Transmission Electron Microscopy (HRTEM). We showed that the U(VI)/bacterium interaction mechanism is highly dependent upon pH but also the nature of the used background electrolyte played a role. At neutral conditions and a U concentration ranging from 5·10^−4^ to 10^−5^ M (environmentally relevant concentrations), XAS analysis revealed that uranyl phosphate mineral phases, structurally resembling meta-autunite [Ca(UO_2_)_2_(PO_4_)_2_ 2–6H_2_O] are precipitated at the cell surfaces of the strain MAH1. The formation of this mineral phase is independent of the background solution but U(VI) luminescence lifetime analyses demonstrated that the U(VI) speciation in seawater samples is more intricate, i.e., different complexes were formed under natural conditions. At acidic conditions, pH 2, 3 and 4.3 ([U] = 5·10^−4^ M, background electrolyte  = 0.1 M NaClO_4_), the removal of U from solution was due to biosorption to Extracellular Polysaccharides (EPS) and cell wall components as evident from TEM analysis. The *L*_III_-edge XAS and TRLFS studies showed that the biosorption process observed is dependent of pH. The bacterial cell forms a complex with U through organic phosphate groups at pH 2 and via phosphate and carboxyl groups at pH 3 and 4.3, respectively. The differences in the complexes formed between uranium and bacteria on seawater compared to NaClO_4_ solution demonstrates that the actinide/microbe interactions are influenced by the three studied factors, i.e., the pH, the uranium concentration and the chemical composition of the solution.

## Introduction

Natural concentrations of uranium in seawater are estimated to be 3 μg/L [1]. The oceans hold billions of tonnes of this radionuclide, almost 1000 times more than is present in terrestrial environments [2]. During the last four decades approximately 34 MCi of uranium has been introduced into the atmosphere by nuclear detonations, the larger fraction of it entering into the sea [3]. In addition, uranium is delivered to the oceans *via* rivers and dust, and is removed by uptake into marine sediments and oceanic basalts [4]. The biogeochemistry of uranium of terrestrial environments has been well studied [5]–[7]. Both abiotic (e.g. minerals, ions, etc.) [8]–[9], and biotic components (microbes, organic matter, etc.) of the terrestrial environment have been shown to mobilize and/or immobilize uranium through different mechanisms including precipitation [10]–[11], biosorption [6], [12], and intracellular accumulation, biotransformation, and chelation [5].

In the case of marine environments, several studies were performed on macroscopic characterization of heavy metal uptake and concentration, and the retention and release of radioactive materials by aquatic organisms [13]–[14]. For instance, the exopolysaccharide produced by the marine bacterium *Enterobacter cloaceae* was reported to have excellent chelating properties with respect to cadmium, copper, and cobalt [13]. Sakamoto et al. [14] determined the concentrations of REEs and U in algal samples taken on the coast of Niigata Prefecture, and suggested that the accumulation mechanism of REEs in brown algae may be different from that of U due to the chemical behavior of the element. However, studies at the molecular level to investigate how exactly, under highly saline conditions, bacteria interact with radionuclides, are still lacking. Such studies are needed to take into account possible U transfer from radioactive waste disposal areas to the open ocean through rivers and estuarine areas [15], i.e. habitats that are characterized by a high microbial diversity [16].

Here we present a multidisciplinary approach on the speciation of uranium sequestered by the marine bacterium *Idiomarina ioihiensis* MAH1, under laboratory and seawater conditions. The strain MAH1 has been biochemically and physiologically characterized, and showed an ability to precipitate a wide variety of minerals including phosphates (e.g. struvite, NH_4_MgPO_4_• 6H_2_O)[17], carbonates (e.g. Ca-Mg kutnahorite, CaMg(CO_3_)_2_) [17], and sulfates (barite, BaSO_4_) in sea water [18]. The characterization of U solid phases associated with the cells of MAH1 using two electrolyte conditions was performed by means of X-ray Absorption Spectroscopy (XAS), Time-Resolved Laser-Induced Fluorescence Spectroscopy (TRLFS), and Transmission Electron Microscopy (TEM). Our aim was to understand the mechanisms by which planktonic marine bacteria interact with uranium in marine environments. The role of these microorganisms in the fate and transport of this radionuclide should be taken into consideration, along with the presence of minerals. The results presented here reveal the potential of marine bacteria for remediating of uranium-containing wastes and for the extraction of uranium from the sea when ground mining would become economically unattractive. In this study we also demonstrated that radionuclide-microbe interactions are best studied under natural habitat conditions.

## Materials and Methods

### Bacterial Growth

The strain MAH1 was isolated from a surface seawater sample of the Alboran Sea, located on the most western corner of the Mediterranean Sea, between the coast of Spain (in the north) and Morocco and Algeria (in the south) [17]. This sea has a strategic position as it connects the Atlantic Ocean to the Mediterranean Sea, and through it passes an intensive maritime traffic. The sampling work was carried out in public seawater and no specific permissions were required for this location. This strain was deposited in the Spanish Type Culture Collection (CECT) (www.cect.org), under reference CECT 5996. The cells were cultured and maintained in marine broth medium (MB)(Difco) [17]; its composition was the following (g/L): NaCl, 19.45; MgCl_2,_ 8.8; peptone, 5; Na_2_SO_3_, 3.24; CaCl_2_, 1.8; yeast extract, 1; KCl, 0.55; NaHCO_3_, 0.16; ferric citrate, 0.1; KBr, 0.08; SrCl_2_, 0.03; H_3_BO_3_, 0.02; Na_2_HPO_4_, 8; Na_2_SiO_3_, 4; NaF, 2.4; NH_4_NO_3_, 1.6. For the preparation of solid media Bacto agar (2 g/L) was added. The bacterial cells were pre-cultured in the above mentioned medium. The bacterial growth was monitored by measuring optical density at 620 nm.

Cells were grown to late exponential-phase under shaking at 30°C, were harvested by centrifugation at 15.000×g for 20 min at 4°C, and washed three times with 0.1 M NaClO_4_ or with Alboran seawater (hereafter referred to as seawater) to remove the interfering ingredients of the growth medium. The cell density used for the uranium/bacterial interaction experiments was 0.4 g dry biomass per liter.

### Chemical Composition of Seawater Solution

Seawater collected from the surface of the Alboran Sea was previously filtered with a 0.2 μm porous membrane immediately after sampling to eliminate particulate material. The chemical composition (in ppm) of seawater used in this study is: Na^+^, 10768; K^+^, 399; Ca^2+^, 412; Mg^2+^, 1291; Cl^−^, 19353; SO_4_^2−^, 2712; HCO_3_^−^, 141; NH_4_^+^, 0.03; NO_3_^−^, 0.29. The speciation of U(VI) in seawater at pH 7.2 and temperature of 25°C was determined using Visual Minteq 3.0 software [19].

### Uranium Solution Preparation

1 M stock solution of UO_2_(NO_3_)_2_ 6H_2_O was prepared by dissolving the appropriate quantity of the metal salt in 0.1 M NaClO_4_. The stock solution was sterilized by filtration through 0.22 μm nitrocellulose filters and was stored at 4°C. Working solutions were prepared by dilution of the stock solution using 0.1 M NaClO_4_ (pH 2, 3, 4.3, and 7) or seawater solutions. The pH was adjusted by addition of small volumes of acid (HCl) or base (NaOH).

### Time-Resolved Laser-Induced Fluorescence Spectroscopy (TRLFS)

For TRLFS measurements, cells of *I. loihiensis* MAH1 were incubated for 48 h in 0.1 M NaClO_4_ at pH 2, 3, and 4.3, each in the presence of 5·10^−4^ M U(VI), as well as at pH 7, in the presence of 1·10^−4^ M U(VI). Parallel cell samples were incubated similarly using seawater (pH 7.2) with following concentrations of U(VI): 1•10^−4^ M, 5•10^−5^ M, and 1•10^−5^ M. After incubation, the cells were washed and suspended in the corresponding background solution (seawater or NaClO_4_). One half of the obtained cell suspension was directly used for spectroscopic measurements. The cells of the remaining suspension were harvested by centrifugation, dried under vacuum and subsequently powdered, according to the XAS sample preparation. About 3 mL of solution or 10 mg of the powdered cell samples were used for the TRLFS measurements. Samples were placed in a quartz micro cuvette. To avoid inhomogeneous liquid samples caused by cell sedimentation all suspensions were stirred during the measurements.

U(VI) luminescence was excited using a Nd YAG laser system (Spectra Physics, Santa Clara, CA, USA) [20] with an excitation wavelength of 410 nm and a low intensity of 300 μJ to avoid sample damage. All measurements were performed at room temperature at the Institute of Resource Ecology, Helmholtz Centre Dresden-Rossendorf, Dresden, Germany. Luminescence spectra were recorded between 454 and 589 nm. The central wavelength of the spectrograph was set to 520 nm and the gate width of the ICCD camera was 1 μs, in case of the solid samples incubated at pH 3 and 4.3, and 5 μs for all other samples (complete detection system: HORIBA Jobin Yvon GmbH, Darmstadt, Germany). For time-resolved measurements a digital delay generator (DG535, Stanford Research Systems, Sunnyvale, CA, USA) was used. Before each series of measurements the background signal was recorded 2 μs after the laser pulse and afterwards automatically subtracted from each spectrum.

The spectrograph was calibrated using a mercury lamp with known emission lines. Luminescence was excited by 50 to 80 laser pulses, depending on the amount of uranium in the sample. Subsequently, 101 U(VI) luminescence spectra (each calculated by averaging three single measurements) were recorded after defined delay times. The obtained luminescence data were processed by using Origin 7.5 software (OriginLab Corporation, Northampton, MA, USA) including the PeakFit module 4.0.

### X-Ray Absorption Spectroscopy Analysis

Samples for XAS studies were prepared as previously described [6]. In the case of seawater samples, the U concentrations assayed, 1•10^−4^ and 2.5 10^−4^ M, were higher than those of the TRLFS studies due to the low sensitivity of this technique to low metal concentrations. After contact with the uranium solution, cells were harvested and washed with either 0.1 M NaClO_4_ or seawater, depending on the experiment. The pellets were dried in an oven at 30°C for 24 h and subsequently powdered.

Uranium *L*_III_–edge X-ray absorption spectra were collected at the Rossendorf Beamline at the European Synchrotron Radiation Facility (ESRF), Grenoble (France) [21] using a Si(111) double-crystal monochromator, and Pt-coated mirrors for focusing and rejection of higher harmonics. Data were collected in fluorescence mode using a 13-element Ge detector (Canberra, Oxford, UK). The energy was calibrated by measuring the yttrium (Y) *K*-edge transmission spectrum of an Y foil and defining the first inflection point as 17038 eV. The biological/uranyl samples were measured as dry samples. The Extended X-ray Absorption Fine Structure (EXAFS) oscillations were isolated from the raw, averaged data by removal of the pre-edge background, and approximated by a first-order polynomial, followed by μ0-removal via spline fitting techniques and normalization using a Victoreen function. Dead-time correction was applied. The ionization energy for the U *L_III_* electron niveau, E0, was arbitrarily defined as 17185 eV for all averaged spectra. The EXAFS spectra were analyzed according to standard procedures using the program EXAFSPAK [22]. The theoretical phase and amplitude functions used in data analysis were calculated with FEFF8 [23] using two different models: i) crystal structure of meta-autunite, Ca(UO_2_)_2_(PO_4_)_2_.6H_2_O [24] for the seawater samples, and ii) a model that contains fragments of two molecules, meta-autunite and uranyl triacetate [6] for the NaClO_4_ samples. FEFF is an automated program for ab initio multiple scattering calculations of EXAFS, X-ray Absorption Near-Edge Structure (XANES) and various other spectra for clusters of atoms. All fits included the four-legged multiple scattering (MS) path of the uranyl group, U-Oax-U-Oax. The coordination number (N) of this MS path was linked to N of the single-scattering (SS) path U-Oax. The radial distance (R) and Debye-Waller factor (σ^2^) of the MS path were linked at twice the R and σ^2^ of the SS path U-Oax, respectively [25]. During the fitting procedure, N of the U-Oax SS path was held constant at two. The amplitude reduction factor (S0^2^) was held constant at 1.0 for the FEFF8 calculation and EXAFS fits. The shift in threshold energy, ΔE0, was varied as a global parameter in the fits.

### HRTEM and Energy Dispersive X-ray (EDX) analyses

Cells incubated with uranium dissolved in 0.1 M NaClO_4_ (uranium concentration 5·10^−4^ M; pH 2, 3 and 4.3) and in seawater (uranium concentration 5·10^−4^ M; pH 7.2) were harvested and cells were fixed in 4% glutaraldehyde in 0.2 M cacodylate buffer with 0.4 M sucrose and 0.1% NaCl (in order to reach an osmolarity of 1205 mOsm similar to the MB medium) for 2 hours at 4°C and then washed three times in the same cacodylate buffer. The cell pellets were then dehydrated with ethanol and embedded in Spurr resin. Next, the samples were thin-sectioned (0.25 μm) using a diamond knife on a Reichert Ultracut S ultramicrotome, and the sections were supported on copper grids and coated with carbon. Samples were examined with a high-resolution Philips CM 200 transmission electron microscope at an acceleration voltage of 200 kV. EDX analysis was also performed at 200 kV using a spot size of 70 Å and a live counting time of 100 s. For selected-area electron diffraction we used a Philips CM 200 transmission electron microscope set in the diffraction mode with a camera length of 1,000 mm and an exposure time between 15 and 20 seconds.

## Results

### Speciation of Uranium in NaClO_4_ and Seawater Solutions

The chemical speciation of U(VI) in the presence of NaClO_4_ or seawater (in the absence of bacterial cells) was determined using Visual Minteq 3.0 software [19]. In the NaClO_4_ system, the dominant species at 5·10^−4^ M U concentration and in the pH range of 2–4.3 is the UO_2_^2+^ ions (Figure S1). At pH 7, U speciation is controlled by (UO_2_)_3_(OH)_5_^+^ (55%) and (UO_2_)_4_(OH)_7_^+^ (45%)(Figure S1). In contrast, in seawater with U concentrations ranging from 5·10^−4^ M to 10^−5^ M, speciation of U(VI) is dominated by Ca_2_UO_2_(CO_3_)_3_ (aq) and CaUO_2_(CO_3_)_3_^2−^ (Figure S2). The results obtained are in agreement with those obtained by Konstantinou and Pashalidis [26] who reported that in surface seawater uranium-carbonate species such as UO_2_(CO_3_)_2_^2−^ and UO_2_(CO_3_)_3_^4−^ are the dominant uranium(VI) species in solution.

### XAS Characterization of U(VI) Coordination by *I. liohiensis* MAH1 Cells in Seawater

XANES spectra (Figure S3) of the uranium complexes formed with cells from strain MAH1 in seawater at U concentrations of 1·10^−4^ and 2.5·10^−4^ M contained a XANES peak at 17,188 eV which has previously been attributed to uranium in the 6+ oxidation state [25].

**Figure 1:**
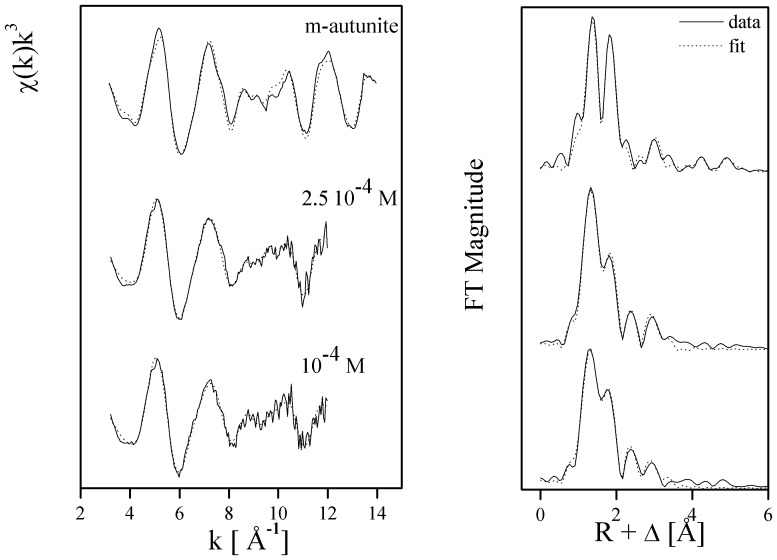
Uranium *L*_III_-edge *k* ^3^–weighted EXAFS spectra (left) and the corresponding Fourier transforms (FT) (right) of the uranium complexes formed by *I. loihiensis* MAH1 cells at U concentrations of 10^−4^ and 2.5 10^−4^ M in seawater, and reference compound (m-autunite).

The uranium *L*_III_-edge EXAFS spectra and their corresponding Fourier transforms (FT) for the uranium species formed at the two U concentrations by the *I. loihiensis* MAH1 cells are plotted in Figure 1. The FT represents a pseudo-radial distribution function of the uranium near-neighbor environment.

The FTs of the EXAFS spectra of the two samples show significant peaks (Figure 1) and the corresponding quantitative fit results are summarized in Table 1 (distances are phase shift corrected). The adsorbed U(VI) has the common linear trans-dioxo structure: two axial oxygen atoms at a radial distance of 1.76–1.77±0.02 Å. A four- to five-fold coordination of uranium (N∼4.4–4.9 and R = 2.27–2.28±0.02 Å) to ligands provided by the bacterial cells was observed on the EXAFS spectra of the two samples. The low Debye-Waller factors obtained (0.0065–0.0073 Å^2^), indicated the absence of a disorder in U-Oeq_1_ distances contributing to the EXAFS signal. Adding an oxygen shell at a distance of R = 2.87–2.90±0.02 Å significantly improved the fit for all samples. However, this shell is not related to direct bonding but has been previously interpreted as scattering contributions from neighboring ligand shells known as “short contacts” in crystallography [10]–[12].

**Table 1:** Structural parameters of the U(VI) complexes formed by *I. loihiensis* MAH1 using seawater as the background electrolyte.

Sample	Shell	N^a^	R(Å)^b^	σ^2^ (Å^2^)^c^	ΔE (eV)
**2.5·10^−4^ M**	U-O_ax_	2^d^	1.77	0.0035	−15.0
	U-O_eq1_	4.4(6)	2.28	0.0065	
	U- O_eq2_	1.0(1)	2.87	0.0038^d^	
	U-P	5.5(5)	3.60	0.0059	
	U- O_eq1_-P (MS)	11.0^e^	3.72^e^	0.0059^e^	
	U-U	1.3(2)	5.26	0.0085	
**10^−4^ M**	U-O_ax_	2^d^	1.76	0.0051	−16.5
	U-O_eq1_	4.9(6)	2.27	0.0073	
	U- O_eq2_	1.3(2)	2.90	0.0038^d^	
	U-P	3.6(4)	3.57	0.0059	
	U- O_eq1_-P (MS)	7.2^e^	3.67^e^	0.0059^e^	
	U-U	1.4(2)	5.17	0.0085	

a Errors in coordination numbers are ±25%, and standard deviations, as estimated by EXAFSPAK are given in parentheses.

b Errors in distance are ±0.02 Å.

c Debye-Waller factor.

d Value fixed for calculation.

e Coordination number linked twice and Debye-Waller factor once to the N and σ^2^ of the U-P path.

The fourth FT peak observed at R+Δ∼3 Å (radial distance R = 3.57–3.60±0.02 Å), is the result of a back-scattering from phosphorus atoms. This distance is typical for a mono-dentate coordination of U(VI) by phosphate [10]–[12]. By plotting the uranium *L*_III_-edge EXAFS spectra of the sample along with the reference meta-autunite we could confirm that uranyl phosphates constitute the main uranium-containing precipitates formed on the bacterial surfaces.

### XAS Characterization of U(VI) Coordination by *I. liohiensis* MAH1 Cells in NaClO_4_ System

As in the case of the seawater samples, XANES analysis of the NaClO_4_ samples (U 5·10^−4^ M, pH values 2, 3 and 4.3) (Figure S3) indicated that the main oxidation state of uranium associated with the bacterial cells was U(VI).

The uranium *L*_III_-edge EXAFS spectra of the three samples were fitted in R-space and the results of the data analysis are summarized in the Table 2 (distances are phase shift corrected). The fit of the different spectra were performed using the model described in Merroun et al. [6] and containing two molecules (m-autunite and uranyl triacetate). This model has been used for the fit of EXAFS spectra of different U(VI)-treated biological samples [6].

**Table 2:** Structural parameters of the uranium complexes formed by *I. loihiensis* MAH1 cells in 5·10^-4^ M using NaClO_4_ as the background electrolyte.

Sample	Shell	N^a^	R(Å)^b^	σ^2^ (Å^2^)^c^	ΔE (eV)
**pH 2**	U-O_ax_	2^d^	1.76	0.0023	−12.0
	U-O_eq1_	4.0(6)	2.33	0.0092	
	U- O_eq2_	1.5(2)	2.90	0.0038^d^	
	U-P	2.2(2)	3.58	0.004	
	U- O_eq1_-P (MS)	4.4^e^	3.69^e^	0.004^e^	
**pH 3**	U-O_ax_	2^d^	1.77	0.0026	−12.0
	U-O_eq1_	3.2(6)	2.35	0.008	
	U- O_eq2_	1.2(2)	2.87	0.0038^d^	
	U-P	1.7(2)	3.59	0.004	
	U- O_eq1_-P (MS)	3.4^e^	3.70^e^	0.004^e^	
**pH 4.3**	U-O_ax_	2^d^	1.77	0.0029	−7.1
	U-O_eq1_	2.3(6)	2.34	0.004	
	U- O_eq2_	1.7(2)	2.51	0.004^e^	
	U-C	0.9(1)	2.89	0.0038	
	U-P	1.3(2)	3.60	0.004	
	U- O_eq1_-P (MS)	2.6^e^	3.70^e^	0.004^e^	

a Errors in coordination numbers are ±25%, and standard deviations, as estimated by EXAFSPAK are given in parentheses.

b Errors in distance are ±0.02 Å.

c Debye-Waller factor.

d Value fixed for calculation.

e Coordination number linked twice and Debye-Waller factor once to the N and σ^2^ of the U-P path.

**Figure 2:**
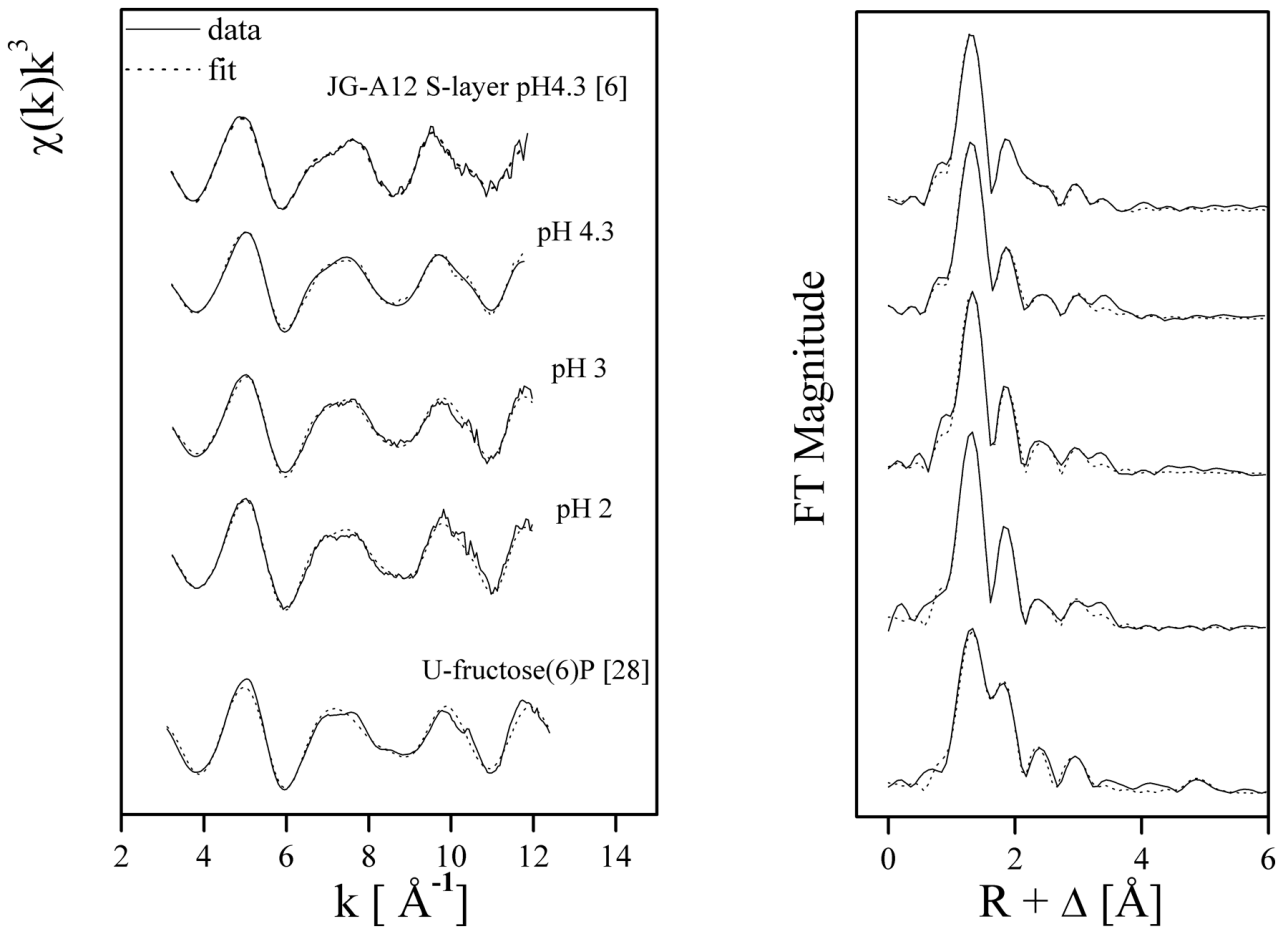
Uranium *L*_III_-edge *k* ^3^–weighted EXAFS spectra (left) and the corresponding Fourier transforms (FT) (right) of the uranium complexes formed by *Idiomarina loihiensis* MAH1 cells at U concentration of 5 10^−4^ M in NaClO_4_, with pH values 2, 3, and 4.3; and reference compounds (U-fructose(6) phosphate [28], uranyl triacetate [6], and surface layer proteins of the *Bacillus sphaericus* strain JG-A12, complexed with U at pH 4.3 [6]).

The different fit results are shown in Figure 2. The derived structural parameters are identical for the two samples treated with U at pH 2 and 3, as already suggested by the similarity of the spectra, and different to those of the sample treated at pH 4.3.

The FTs of the EXAFS spectra of the uranium-bacterial cell complexes show four to five significant peaks. In all samples, fitting demonstrated that U(VI) has two O_ax_ at a distance of 1.76 to 1.77 Å. The samples incubated at pH 2 and 3, show the presence of a four-fold uranium coordination (N∼4 and R = 2.33–2.35±0.02 Å).

In the case of the sample treated at pH 4.3, the Oeq shell is split into two components, with the first component (U-Oeq1) at a distance of 2.34 Å and the second component (U-Oeq_2_) at a somewhat longer distance (2.51 Å). These two shells could not be represented as separate peaks in the FT. The U-Oeq1 bond distance is within the range of previously reported values for the phosphate oxygen atom bound to uranyl [10]–[12]. The longer equatorial oxygen bond length of 2.51±0.02 Å is similar to previously reported values for the carboxyl oxygen atom bound to uranyl (2.45 to 2.51 Å) [27]. The EXAFS spectrum of the sample at pH 4.3 is similar to that of U(VI) complexes formed by the S-layer proteins of *Bacillus sphaericus* JG-A12, where U(VI) is coordinated to phosphate groups in a monodendate binding mode and to carboxyl groups in a bidendate binding fashion [6].

In all samples a peak at a bond distance between 3.58 and 3.60 Å was fitted, which corresponds to the contribution of phosphate groups in the coordination of U(VI) in a monodendate binding mode.

### Time-Resolved Laser-Induced Fluorescence Spectroscopy Analysis

**Figure 3:**
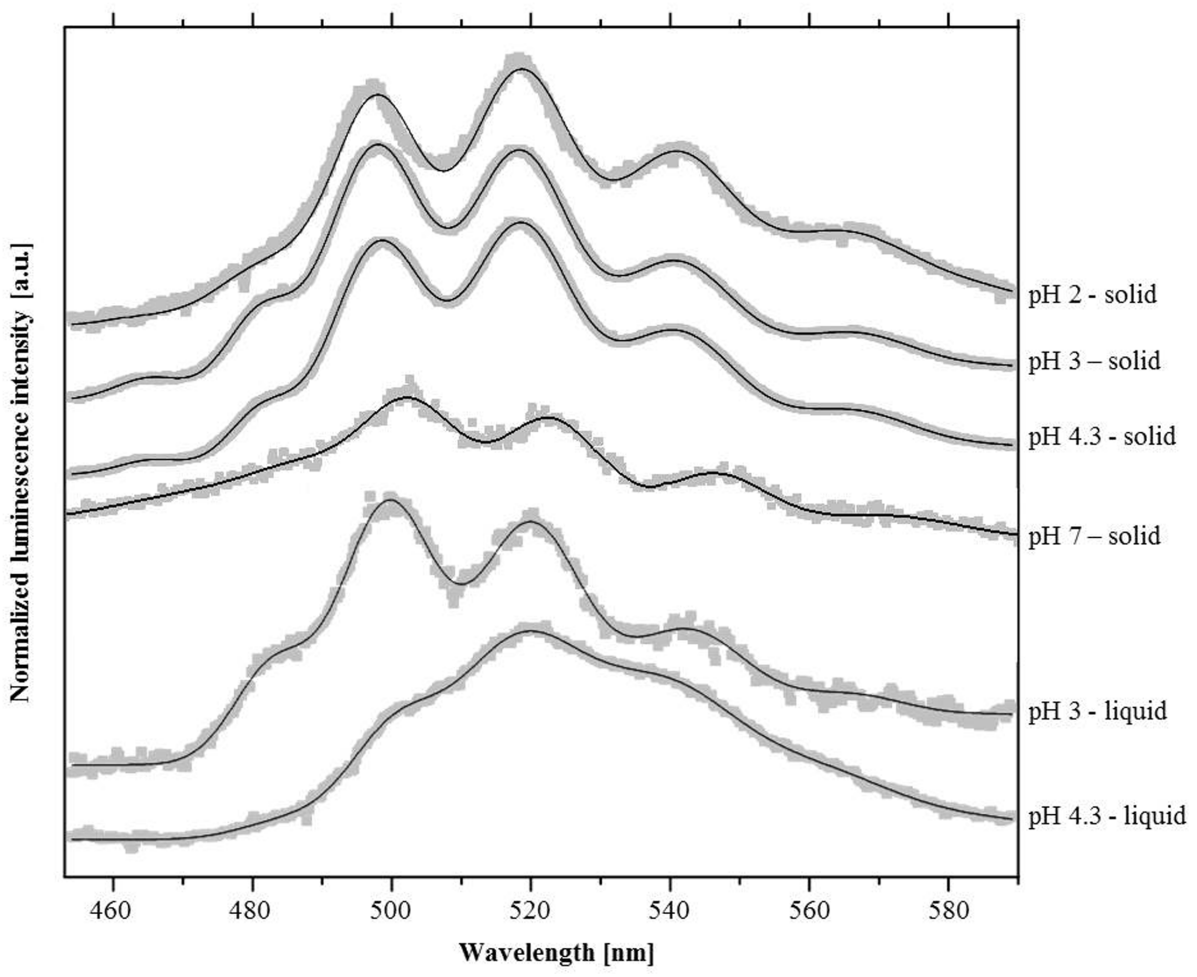
Luminescence spectra of the U(VI) complexes formed by the marine bacterium *I. loihiensis* MAH1 in NaClO_4_ background solution. Samples were measured in form of dried powder and directly within the background solution.

Time-resolved laser-induced fluorescence spectroscopy is a technique that allows one to determine the U(VI) speciation, even in highly complex biological systems. The luminescence spectra recorded from *I. loihiensis* MAH1 cells, incubated with 5·10^−4^ M U(VI) at pH 2, pH 3, pH 4.3, and pH 7 (10^−4^ M of U) in 0.1 M NaClO_4_ solution, are shown in Figure 3. The corresponding luminescence emission maxima are summarized in Table 3.

**Table 3:** Luminescence emission maxima of the U(VI) complexes formed by *I. loihiensis* MAH1 cells in NaClO_4_ and selected uranyl model complexes.

Sample	Luminescence emission maxima (nm)^a^^,^ b				Lifetime(s) (μs)
**NaClO_4_ samples**					
*I. loihiensis* - pH 2 (solid)		**497.5**	**518.1**	**540.9**	
*I. loihiensis* - pH 3 (solid)	465.4	**497.7**	**518.2**	**540.6**	
*I. loihiensis* - pH 4.3 (solid)	465.2	**498.2**	**518.3**	**540.8**	Table S1
*I. loihiensis* - pH 7 (solid)		**502**	**522.6**	**545.9**	
*I. loihiensis* - pH 3 (liquid)		**499.4**	**519.8**	**541.4**	
*I. loihiensis* - pH 4.3 (liquid)		**499.9**	**517.8**	**538.9**	
**Reference samples**					
**Organic uranyl phosphate complexes**					
UO_2_-fructose(6)phosphate [28]		**497.1**	**519.0**	**543.3**	0.13±0.05
UO_2_-AMP [29]		**497**	**519**	**542**	n.d.
R-O-PO_3_-UO_2_ [30]		**498.1**	**519.6**	**542.9**	1.2±0.4
**Organic uranyl carboxylate complexes**					
(R-COO)_2_-UO_2_ [35]	466.0	**498.1**	**518**	**539**	0.7±0.1
R-COO-UO_2_^+^ [35]					7.3±1.4
**UO_2_^2+^ and hydrolytic species**					
UO_2_^2+^ (pH 1.5)		**489.5**	**511**	**534.3**	1.92±0.12
UO_2_OH^+^ [48]		**497.3**	**518.4**	**541.3**	32.8±2.0
(UO_2_)_3_(OH)_5_^+^ [49]		**496**	**514**	**535**	23±3
(UO_2_)_2_(OH)_2_^2+^ [48]		**498.3**	**519.7**	**543.4**	9.5±0.3

a Main luminescence emission bands were pointed out by bold letters.

b Error of emission bands is ±0.5 nm.

The luminescence properties of the cell sample incubated at pH 2 are characteristic of U(VI) complexes formed at phosphate groups of organic molecules such as sugar phosphates including fructose phosphates [28], phosphorylated nucleosides (e.g. ATP) [29], or lipopolysaccharides of the gram-negative bacterium *E. coli* [30]. At this pH, TEM analysis indicated that it is likely that these macromolecules were liberated by cell lysis induced by exposure to such low pH values. This is in agreement with other U(VI)-microbe complexation studies at corresponding pH conditions reporting that phosphate groups are the main U binding sites [29], [31]–[32]. Similar spectroscopic results were also obtained for the solid samples incubated at pH 3 and pH 4.3 (Figure 3, Table 3), indicating that uranyl phosphate complexes were formed at these pH conditions as well. However, in both samples an additional luminescence peak around 465 nm was observed, which could not be assigned to uranyl phosphate complexes. Luminescence peaks at similar wavelength were described for uranyl carboxylate complexes formed at the surface of bacterial [33] or algal cells [34], as well as with purified cellular compounds, e.g. peptidoglycan [35]. While this complexation behaviour of U(VI) might be expected at pH 4.3 because carboxylic groups of bacterial cell walls start to deprotonate around this pH and thus acquire the ability to bind (heavy) metals [36], the formation of uranyl carboxylate complexes at pH 3 is rather unusual to be considered for microbe/uranium interactions. Nonetheless, some carboxyl acids exhibited a pKa close to 2 [37] suggesting that strain-specific cell surface compounds may still provide active U(VI) binding sites at extreme acidic conditions i.e. as low as pH 2. Time-resolved analyses of the samples revealed a tetra-exponential decay of the U(VI) luminescence, indicating a mixture of at least four different fluorescent uranium species, respectively (Table S1).

The four luminescence lifetimes of the solid samples (pH 2, 3 and 4.3) are highly comparable to each other with average values of about 1 μs, 5.6 μs, 26 μs and 98 μs (Table S1). Fitting procedures at different delay times showed no shift of the luminescence emission maxima, indicating a high structural similarity of the formed complexes.

In contrast to the acidic samples, we detected at pH 7 only one uranium species with a luminescence lifetime of 41±2 μs (Table S1). Moreover, we observed a bathochromic shift, i.e. shift to a longer wavelength, of the U(VI) spectrum (Figure 3). The main luminescence peaks were located at 502 nm, 522.6 nm, and 545.9 nm and are very similar to those reported for various inorganic uranyl phosphate and uranyl carboxylate compounds and minerals (Table 4).

**Table 4:** Luminescence emission maxima of the U(VI) complexes formed by *I. loihiensis* MAH1 cells in seawater and selected uranyl model complexes.

Sample	Luminescence emission maxima (nm)^a^^,^ b				Lifetime(s) (μs)
**Seawater samples**					
*I. loihiensis* - [UO_2_^2+^] = 1•10^−5^ M (solid)		**502.2**	**522**	**544.6**	
*I. loihiensis* - [UO_2_^2+^] = 5•10^−5^ M (solid)		**502.5**	**522.6**	**544.7**	
*I. loihiensis* - [UO_2_^2+^] = 1•10^−4^ M (solid)		**503.6**	**524.2**	**546.3**	Table S1
*I. loihiensis* - [UO_2_^2+^] = 1•10^−5^ M (liquid)		**503.8**	**523**	**543.1**	
*I. loihiensis* - [UO_2_^2+^] = 5•10^−5^ M (liquid)		**502.7**	**523.4**	**545.2**	
*I. loihiensis* - [UO_2_^2+^] = 1•10^−4^ M (liquid)		**502.8**	**523.4**	**545.9**	
**Reference samples**					
**Inorganic phosphates**					
UO_2_PO_4_^−^ [50]		**502.2**	**524**	**548**	n.d.
(UO_2_)_x_(PO_4_)_y_ [51]		**503**	**523.7**	**546.9**	n.d.
**Uranyl carbonate minerals**					
Ca_2_[UO_2_(CO_3_)_3_] 10 H_2_O [52]	465.4	**502.7**	**524.5**	**545.5**	145±5
Ca_2_(UO_2_)(CO_3_)_3_ 11 H_2_O [52]	466.9	**502.7**	**524.1**	**545.9**	313±10
Ca_2_[UO_2_(CO_3_)_3_] in solute [53]	465	**504**	**524**		0.04±0.01
**Uranyl phosphate minerals**					
Autunite [54]		**504**	**524.2**	**548**	5.15±0.28
Meta-autunite [54]		**501.8**	**522.9**	**546.9**	0.74±0.1
Mg[UO_2_PO_4_]_2_ 10 H_2_O [54]		**501.1**	**522.1**	**545.7**	2.25±0.2

a Main luminescence emission bands were pointed out by bold letters.

b Error of emission bands is ±0.5 nm.

The spectra of the liquid samples showed different luminescence properties compared to the solid samples. At pH 3, the emission maxima of the liquid samples were shifted more than 1 nm to higher wavelength values. This shift was most likely caused by highly luminescent hydrolytic species, especially (UO_2_)_2_(OH)_2_^2+^, which are formed in the solution. At pH 4.3, the influence of these hydrolytic species on the luminescence spectrum is even more pronounced (Figure 3) and their luminescence mask the luminescence of U(VI) complexes formed by the cells. As a result, analysing liquid samples by TRLF has some disadvantages, since a clear U speciation cannot be achieved owing to interferences. On the contrary, performing these analyses in solid samples show no interferences.

In order to recreate conditions similar to the natural habitat of strain MAH1, we additionally investigated the speciation of the U(VI) complexes formed by this bacterium using seawater as the background solution. In this approach the cells were treated with 10^−4^ M, 5•10^−5^ M, and 10^−5^ M U(IV). It is known that luminescence of the uranyl ion in aqueous solution is quenched by halide ions, which are present in large concentrations in seawater. Indeed, we found that the luminescence of the uranium is completely quenched in seawater (data not shown). A corresponding quenching effect of more than 50% was also observed during the measurements of the U(VI)/bacteria in seawater samples.

**Figure 4:**
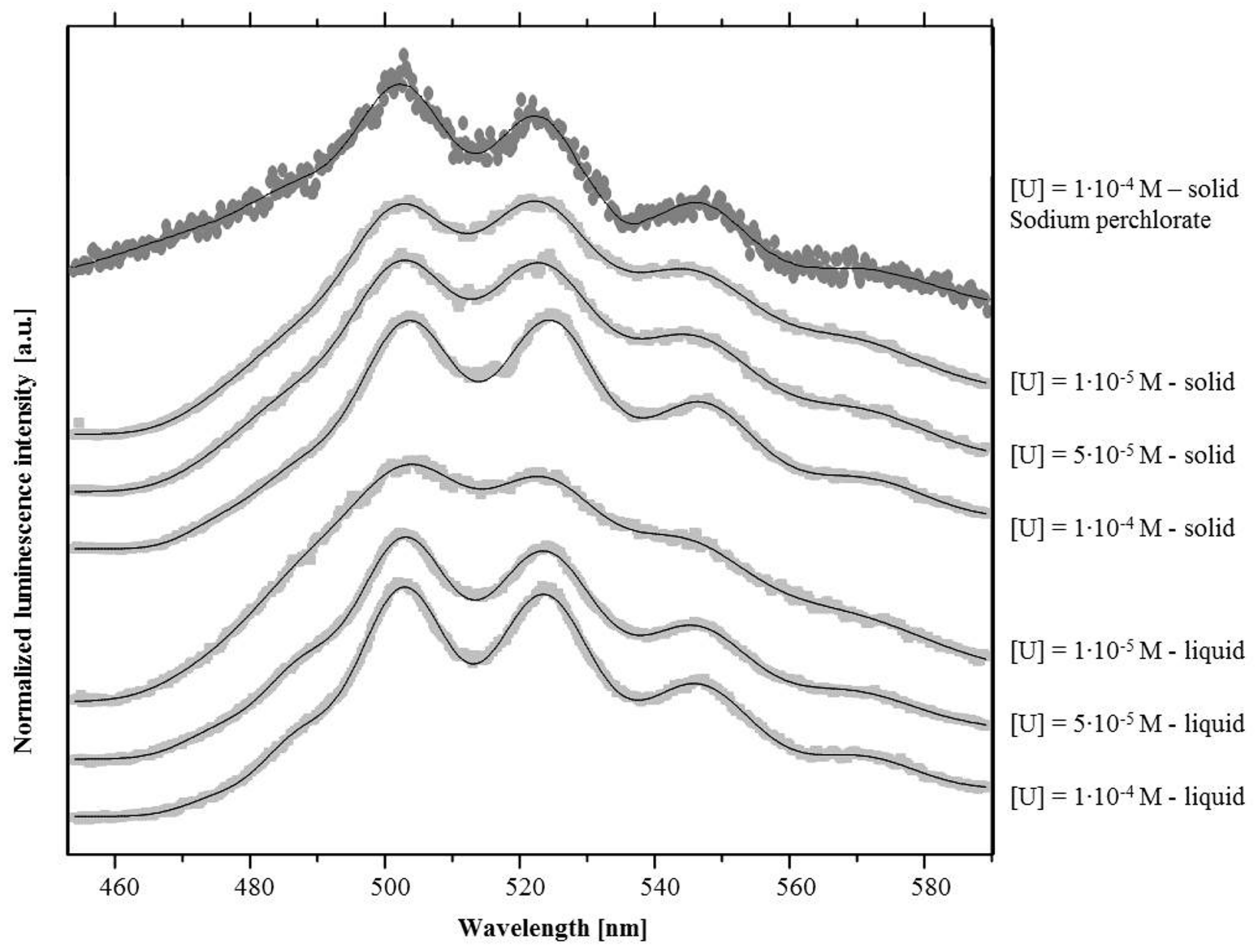
Luminescence spectra of the U(VI) complexes formed by the marine bacterium *I. loihiensis* MAH1 at neutral pH in 0.1 M NaClO_4_ and in seawater background solution. Samples were measured in either dried powder form or directly within the background solution (see text).

The U(VI) luminescence properties of the dried bacteria in seawater samples at different U concentrations are highly comparable to each other (Figure 4, Table 4). Compared to the samples described above, the emission maxima are consistently located at higher wavelengths. Moreover, an additional bathochromic shift was observed with increasing U(VI) concentrations. However, the luminescence peaks of the liquid sample at 10^−5^ M U(VI) are less distinct and merge with each other which hinders proper peak localization (Figure 4). The poor signal is caused by the low uranium concentration combined with the quenching effects of the high halide concentration of the seawater.

The U(VI) main emission maxima of the cell samples incubated in seawater are comparable to those of the sample incubated in 0.1 M NaClO_4_ at pH 7. Thus, they are similar to those reported for various inorganic uranyl phosphate and uranyl carboxylate compounds and minerals (Table 4), and also to those of U precipitates produced by decomposed cells of *Bacillus sphaericus* [31]. This indicates that the strain MAH1 precipitates U(VI) as uranyl phosphate mineral phases. In contrast to the sample incubated in sodium perchlorate solution where only one uranium species was found, the best fit of the U(VI) luminescence decay was obtained in seawater samples using a tri-exponential decay function. However, we again did not observe any significant shifts of the luminescence emission maxima. The obtained luminescence lifetimes of the U(VI) complexes obtained from the bacteria solid samples and the bacteria liquid samples are very comparable. According to the literature, the shorter lifetimes may be assigned to uranyl phosphate mineral phases, whereas the longer lifetime could also result from a low amount of the more stable uranyl carbonate mineral phases.

### TEM/EDX Analysis

**Figure 5:**
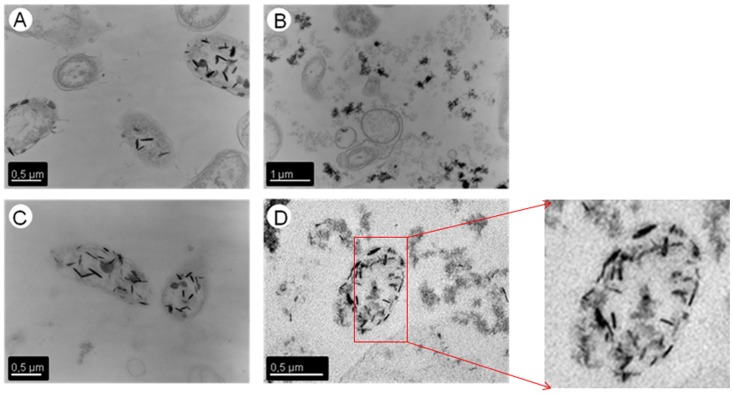
Transmission electron micrographs of thin sections of U-treated *I. loihiensis* MAH1 cells (U at 5•10^−4^ M; in 0.1 M NaClO_4_) at pH 3 (A), and pH 4.3 (B, C, D).

**Figure 6:**
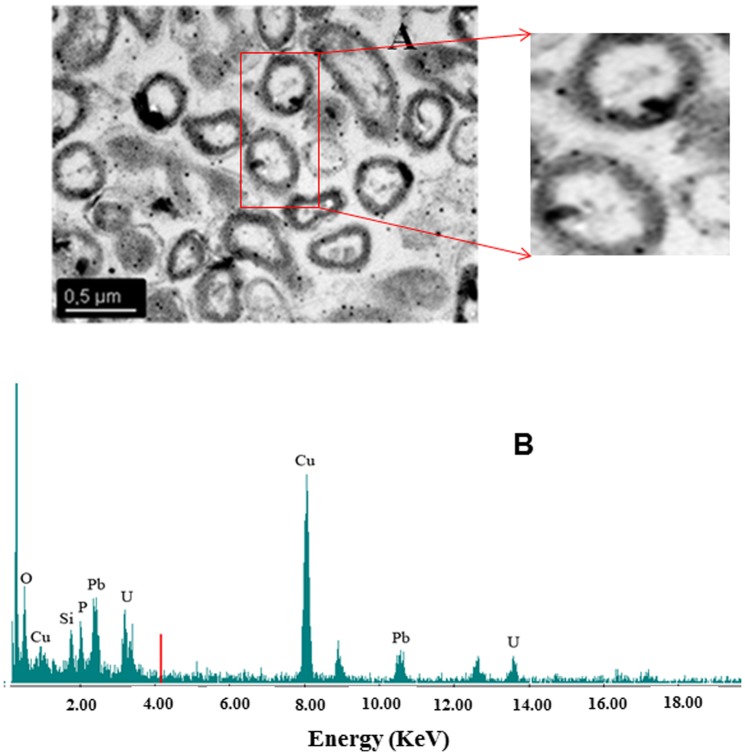
Transmission electron micrograph of a thin section of U-treated *I. loihiensis* MAH1 cells (U at 5•10^−4^ M; in seawater) (A). Energy Dispersive X-ray spectrum of U accumulates indicated by black arrow (B). Uranium is accumulated mainly at the cell surface. No intracellular uptake of this radionuclide was observed.

TEM micrographs of thin sections of MAH 1 cells exposed to 5•10^−4^ M U(VI) at pH values of 3 and 4.3 are shown in Figure 5. At these pH values, the U is localized mainly in the cytoplasm and at the cell surface. In addition, U accumulation within the EPS was shown for the sample at pH 4.3. The EDX spectra of the accumulated U displayed X ray emission peaks corresponding to U and P (Figure S4). No results were obtained at pH 2 due to the loss of cell integrity at this pH value. In seawater and at a uranium concentration of 5•10^−4^ M, TEM analyses demonstrated that the accumulated uranium was located mainly at the cell wall (Figure 6).

## Discussion

Although fundamental chemical and physical characteristics of marine environments have been established [38], the microbial processes in these environments are now being investigated using cell biology and genomics [39]. In particular, information about the interaction mechanisms of marine microorganisms with actinides such as uranium is sparse. Microorganisms from marine ecosystems may, similar to terrestrial microorganisms, sequester uranium through mechanisms such as biosorption [6], biomineralization [10], intracellular accumulation [40], and biotransformations [5]. Here, we studied the interactions of U with the MAH1 strain of *I. loihiensis*, under seawater conditions and under acidic conditions using NaClO_4_ as a background electrolyte.

### U/Bacterial Interactions under Seawater Conditions

In seawater, under environmentally relevant conditions, MAH1 cells precipitated U(VI) as uranyl phosphates with a structure similar to that of meta-autunite. In the absence of U(VI) and under seawater conditions, we previously reported [17] that this marine bacterium has a capacity to biomineralize phosphate and carbonate compounds. In that study we also showed that *I. loihiensis* MAH1 cells are able to precipitate struvite (NH_4_MgPO_4_• 6 H_2_O) crystals that appear encased by small Ca-Mg kuntharonite (CaMg(CO_3_)_2_) spheres and dumbbells and that the proportion of the phosphate and carbonate phases produced by this bacterium amounted to circa 80% and 20%, respectively.

Regarding the meta-autunite biomineralization potential of strain MAH1, it is likely that microbial cell surfaces provide sites for the formation of U phosphates. Phosphate groups responsible for the U binding are originated from cell membranes and/or EPS. Previous studies postulated that formation of nucleation sites on the cell surface is important for initiation of metal precipitation, and that membrane phospholipids may function as sites for uranyl phosphate crystallization [41]–[42]. Our High-Resolution Transmission Electron Microscopy analyses are consistent with this process (Figure 6), showing the cell surface localization of the accumulated U. However, we have no information on how bacteria participate, actively or passively, in the precipitation of U phosphates. Two possible mechanisms are considered: 1) bacteria are playing an indirect passive role in the biomineralization of U(VI) phosphates providing necessary binding sites for this radionuclide (through availability of EPS and/or direct attachment to cell walls), increasing its concentration locally, 2) alkaline phosphatase activity could be involved in the degradation of dissolved organic phosphates (DOP) of seawater helping precipitation of U phosphates. However, we have no evidence of any active mechanism for the U precipitation since we used U solutions without any nutrients to support the metabolic activity of the cells.

To our knowledge this is the first study that demonstrates the ability of marine bacteria to form, in seawater, U(VI) phosphate phases with a structure similar to that of meta-autunite. The *in-situ* biomineralization of U phosphates by biofilm-forming microorganisms in natural terrestrial environments has been described recently by Krawczyk-Baersch et al. [43] using EF-TEM/EELS. The U biomineralization via phosphates is considered to be an efficient process of U immobilization in marine environments, and U phosphates precipitated by microbes are considered insoluble and stable, thus providing a long-term sink for uranium [44].

### Interaction of the Strain MAH1 with U(VI) in 0.1 M NaClO_4_ at Acidic conditions

The speciation of U(VI) in solution at 5•10^−4^ M (at a pH of 2, 3 and 4.3) in 0.1 M NaClO_4_ is dominated by uranyl ion (UO_2_^2+^) known as a highly soluble, mobile and toxic U species. Upon addition of bacterial biomass to the U solution, EXAFS and TRLFS analyses (Figure 2 and Figure 3, respectively) revealed that biosorption is the main interaction process involved. This is a pH-dependent process mediated by cell surface phosphate and carboxyl groups.

EXAFS analysis indicated that at pH 2 and 3, the U(VI) is coordinated by organic phosphate groups in a monodendate binding mode where the local coordination of U(VI) in the corresponding complexes resembles much that of those of U(VI) complexed by fructose-6P [28]. However, at pH 4.3 the cells additionally sequester U(VI) through carboxyl groups in a bidendate binding fashion. These carboxyl groups are probably located within the peptidoglycan layer.

The molecular scale investigation on the pH-dependent U sorption by bacteria and their cell wall components has been well documented. For instance, Kelly et al. [45] showed that both phosphate and carboxyl groups participate in the binding of U(VI) by the cell walls of *Bacillus subtilis*, where an increase of carboxyl and a decrease of phosphoryl coordination was noted with increasing pH from 1.7 up to 4.8. Another type of pH-dependent U(VI) coordination on bacteria (e.g. *Stenotrophomonas* sp.) isolated from uncontaminated and heavy metal-contaminated environments was reported by us previously [46] at acidic conditions. In these studies, organic phosphate groups were the main binding sites for U(VI) at pH 2 and 3. At pH 4.5, the cells precipitated U(VI) as uranium phosphates mineral phases with structures similar to that of autunite/meta-autunite.

We postulate that the phosphate groups involved in the coordination of U(VI) by *I. loihiensis* MAH1 cells at three acidic pH values are likely derived from phospholipids of the cytoplasmic membrane and from lipopolysaccharides of the outer membrane. This hypothesis is supported by TEM analysis which showed that U(VI) accumulated by strain MAH1 is located mainly at the cell wall and within the EPS of this bacterium. We have found some intracellular accumulates which could be due to the loss of cell membrane integrity as a consequence of the low pH values used. It was previously shown by us that the cell viability of this strain is reduced to about 5 to 10% at pH values lower than 4.3 [47].

## Conclusions

The results of the present study show clearly that the speciation of uranium associated with *I. loihiensis* strain MAH1 depends mainly on the pH, although also the uranium concentration and the presence of a background electrolyte are important factors. In the sodium perchlorate (NaClO_4_) system and under acidic conditions, the cells form U-phosphate and/or U-carboxylate complexes. In contrast, at neutral conditions, U-phosphate phases are precipitated by MAH1 cells in both seawater and sodium perchlorate solutions, although U(VI) luminescence lifetime analyses demonstrated that the U(VI) speciation in seawater follows a more intricate process. Taken together, we suggest that, in order to understand the microbial effects on the mobility and transport of radionuclides in marine environments, the experimental set-up should mimic the natural conditions as close as possible. With this study we demonstrate for the first time that marine bacteria have the ability to form, in seawater, U(VI) phosphate phases with a structure that resembles meta-autunite.

## Supporting Information

Figure S1 **Uranium speciation in 0.1 M NaClO_4_, U concentration of 5·10^−4^ M, 25°C.**
(TIF)

Figure S2 **Uranium speciation in seawater at U concentrations of 5·10^−4^ M (A), 2.5·10^−4^ M (B), 1·10^−4^ M (C), 5·10^−5^ M (D), 10^−5^ M (E), pH 7.2, 25°C.**
(TIF)

Figure S3 **Normalized uranium L_III_-edge XANES spectra of 0.04 M U(IV) in 1 M HClO_4_, 0.04 M U(VI) in 1 M HClO_4_, uranium complexes formed by the cells of the strain MAH1 at different experimental conditions: 5·10^−4^ M U in 0.1 M NaClO_4_, (A) pH 2; (B) pH 3; (C) pH 4,3; and (D) 2.5·10^−4^ M U in seawater; (E) 10^−4^ U in seawater.** The spectra were normalized to equal intensity at 17230 eV.
(TIF)

Figure S4 **EDX spectrum of accumulate U located in the interior of the U-treated cells (5·10^−4^ M U in 0.1 M NaClO_4_, pH 4.3).**
(TIF)

Table S1 **Luminescence lifetimes calculated from room temperature TRLF spectroscopic measurements of the U(VI) complexes formed by** ***I. loihiensis*** **MAH1 at different experimental conditions.**
(DOC)
