# Supplementary figures and images for: Biosorption and Biomineralization of U(VI) by the Marine Bacterium Idiomarina loihiensis MAH1: Effect of Background Electrolyte and pH
Source: PLoS One. 2014 Mar 11;9(3):e91305. doi: 10.1371/journal.pone.0091305 (PMC3949747; doi:10.1371/journal.pone.0091305)

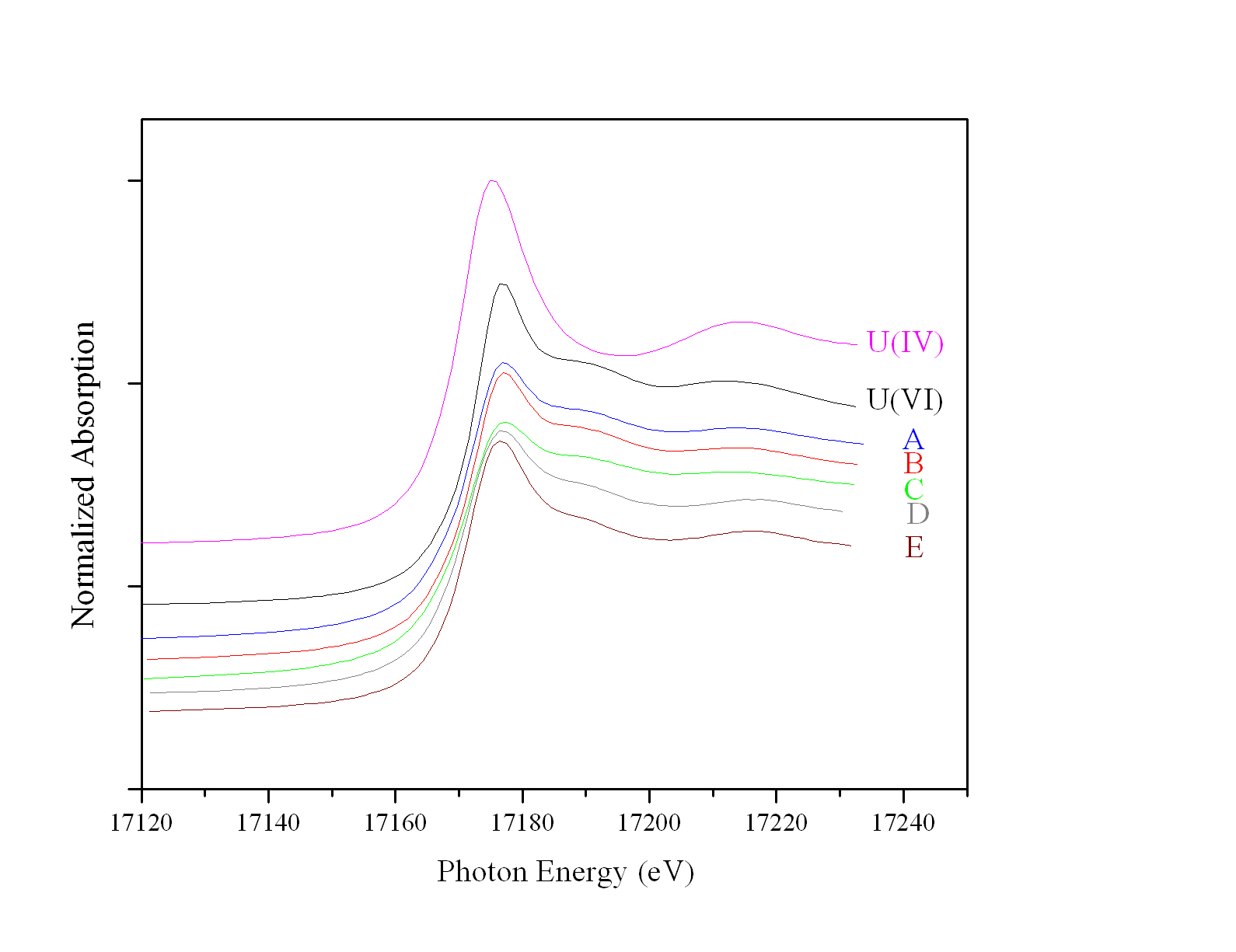

Supplement: Figure S3 — Normalized uranium LIII-edge XANES spectra of 0.04 M U(IV) in 1 M HClO4, 0.04 M U(VI) in 1 M HClO4, uranium complexes formed by the cells of the strain MAH1 at different experimental conditions: 5·10−4 M U in 0.1 M NaClO4, (A) pH 2; (B) pH 3; (C) pH 4,3; and (D) 2.5·10−4 M U in seawater; (E) 10−4 U in seawater. The spectra were normalized to equal intensity at 17230 eV. (TIF) [file pone.0091305.s003.tif]

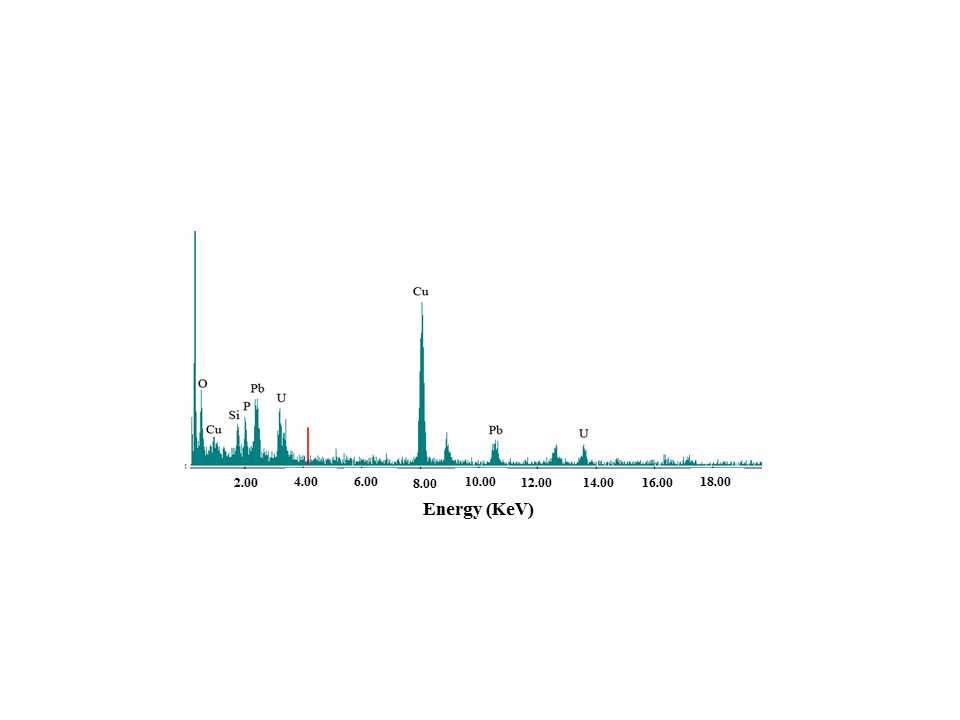

Supplement: Figure S4 — EDX spectrum of accumulate U located in the interior of the U-treated cells (5·10−4 M U in 0.1 M NaClO4, pH 4.3). (TIF) [file pone.0091305.s004.tif]
