# Supplementary material for: Biosorption and Biomineralization of U(VI) by the Marine Bacterium Idiomarina loihiensis MAH1: Effect of Background Electrolyte and pH
Source: PLoS One. 2014 Mar 11;9(3):e91305. doi: 10.1371/journal.pone.0091305 (PMC3949747; doi:10.1371/journal.pone.0091305)
Supplement: Table S1 — Luminescence lifetimes calculated from room temperature TRLF spectroscopic measurements of the U(VI) complexes formed by I. loihiensis MAH1 at different experimental conditions. (DOC) [file pone.0091305.s005.doc]

SUPPORTING MATERIAL

**Biosorption and biomineralization of U(VI) by the marine bacterium *Idiomarina loihiensis*** MAH1**: effect of background electrolyte and pH**

Fernando Morcillo^1^, María T. González-Muñoz^1^, Thomas Reitz^2a^, María E. Romero González^3^, José M. Arias^1^, Mohamed L. Merroun^1*^

^1^Departamento de Microbiología, Universidad de Granada, Granada, Spain

^2^Institute of Resource Ecology, Helmholtz-Zentrum Dresden-Rossendorf, Dresden, Germany

^3^Structural and Civil Engineering Department, University of Sheffield, Sheffield, UK

*Corresponding author: merroun@ugr.es tel: + 34 958 249331, fax: + 34 958 249486

Present address

^a^Department of Soil Ecology, Helmholtz Centre for Environmental Research – UFZ, Halle, Germany

Key word marine bacterium, U(VI), TRLFS, XAS, TEM, EDX

**Table S1.** Luminescence lifetimes calculated from room temperature TRLF spectroscopic measurements of the U(VI) complexes formed by *I. loihiensis* MAH1 at different experimental conditions.

| **Sample** | **Luminescence Lifetimes (µs)** | | | |
| --- | --- | --- | --- | --- |
| **NaClO_4_ samples** | **Lifetime 1** | **Lifetime 2** | **Lifetime 3** | **Lifetime 4** |
| *I. loihiensis* - pH 2 (solid) | 0.8 ± 0.1 | 5.5 ± 0.3 | 27 ± 6 | 103 ± 9 |
| *I. loihiensis* - pH 3 (solid) | 1.1 ± 0.1 | 5.6 ± 0.4 | 23 ± 1 | 98 ± 2 |
| *I. loihiensis* - pH 4.3 (solid) | 1.0 ± 0.1 | 5.8 ± 0.3 | 27 ± 1 | 94 ± 4 |
| *I. loihiensis* - pH 7 (solid) |  |  | 41 ± 2 |  |
| *I. loihiensis* - pH 3 (liquid) | 2.4 ± 0.2 | 14 ± 1 | 70 ± 18 |  |
| *I. loihiensis* - pH 4.3 (liquid) | 6.9 ± 0.3 | 26 ± 2 |  |  |
| **Seawater samples** |  |  |  |  |
| *I. loihiensis* - [UO_2_^2+^] = 1∙10^-5^M (solid) | 5.4 ± 0.5 | 36 ± 3 | 168 ± 16 |  |
| *I. loihiensis* - [UO_2_^2+^] = 5∙10^-5^M (solid) | 9.5 ± 0.6 | 50 ± 6 | 172 ± 13 |  |
| *I. loihiensis* - [UO_2_^2+^] = 1∙10^-4^M (solid) | 7.3 ± 1.2 | 44 ± 4 | 161 ± 5 |  |
| *I. loihiensis* - [UO_2_^2+^] = 1∙10^-5^M (liquid) | 1.9 ± 0.3 | 11 ± 1 | 72 ± 11 |  |
| *I. loihiensis* - [UO_2_^2+^] = 5∙10^-5^M (liquid) | 2.2 ± 0.3 | 12 ± 1 | 52 ± 17 |  |
| *I. loihiensis* - [UO_2_^2+^] = 1∙10^-4^M (liquid) | 2.5 ± 0.1 | 14 ± 1 | 74 ± 27 |  |
